# Nutrition Education in Internal Medicine Residency Programs and Predictors of Residents’ Dietary Counseling Practices

**DOI:** 10.1177/2382120518763360

**Published:** 2018-03-21

**Authors:** Stutee Khandelwal, Sarah E Zemore, Anke Hemmerling

**Affiliations:** 1 School of Public Health, University of California, Berkeley, Berkeley, CA, USA; 2 Fresno Medical Education Program, Department of Medicine, University of California, San Francisco, Fresno, CA, USA; 3 Alcohol Research Group, Public Health Institute, Emeryville, CA, USA

**Keywords:** Nutrition education, residency, dietary counseling, Internal Medicine

## Abstract

**Background:**

Although physicians are expected to provide dietary counseling for patients with cardiovascular (CV) risk factors such as hypertension, hyperlipidemia, diabetes, and obesity, nutrition education in graduate medical education remains limited. Few studies have recently examined nutrition education and dietary counseling practices in Internal Medicine (IM) residency training.

**Objectives:**

To conduct a contemporary assessment of outpatient nutrition education in IM residency programs in the United States, identify predictors of residents’ dietary counseling practices for CV risk factors, and identify barriers for educators in providing nutrition education and barriers for residents in counseling patients.

**Design:**

Cross-sectional anonymous surveys were completed by IM program directors (PDs) and residents throughout the United States. Linear regression was used to examine the association between the amount of nutrition education received and the number of instruction methods used by the residents and frequency of residents’ dietary counseling for patients with CV risk factors.

**Key Results:**

A total of 40 educators (PDs and ambulatory/primary care PDs) and 133 residents across the United States responded to the survey. About 61% of residents reported having very little or no training in nutrition. Nutrition education in residency, both the amount of education (β = 0.20, *P* = .05) and the number of instruction methods used (β = 0.26, *P* = .02), predicted frequency of residents’ dietary counseling practices independent of nutrition education in medical school, which was also significantly associated with counseling (β = 0.20, *P* = .03). Residents’ total fruit and vegetable intake likewise predicted frequency of counseling (β = 0.24, *P* < .001). Low perceived faculty expertise was a major barrier for educators and was associated with lower level of provided nutrition education (*r* = −.33, *P* = .04). Low resident and low perceived clinic preceptors’ interests in nutrition were also associated with lower frequency of residents’ dietary counseling (*r* = −.19, *P* = .04; *r* = −.18, *P* = .05).

**Conclusions:**

The provision of nutrition education in IM residency programs and IM residents’ dietary counseling for patients need to be systematically assessed nationally. This study’s preliminary findings suggest that multimodal nutrition education in IM residency and better resident dietary habits are associated with higher frequency of dietary counseling for patients. Lack of faculty expertise and low faculty and resident interests in patient counseling need to be addressed perhaps by mandating nutrition education in graduate and continuing medical education.

## Background

The US Preventive Services Task Force and the American College of Cardiology/American Heart Association recommend dietary intervention for diet-related cardiovascular (CV) risk factors such as hypertension, hyperlipidemia, diabetes, and obesity.^1,2^ Yet, rates of dietary counseling by physicians are generally low, ranging from 25% to 40% of primary care visits.^3–5^ Moreover, physicians consistently report inadequate training in nutrition and believe that better training would improve their patient care, underscoring the importance of further research in this area.^3,6^

Internal Medicine (IM) is the largest specialty that trains physicians in adult chronic disease management and accounts for 24% of US physician residency positions.^7^ However, nutrition education is not explicitly mentioned under the Accreditation Council for Graduate Medical Education (ACGME) program requirements for IM.^8^ Only 2 older studies from the early 1990s which examined the provision of nutrition education across 7 medical specialties included educators from IM programs.^9,10^ Given that each residency specialty has different curricula, priorities, and ACGME requirements, results from one specialty may be not applicable to another. Later studies found that 94% of first-year IM residents felt that dietary counseling was their obligation, but only 14% felt that physicians were adequately trained in this area.^11^ Furthermore, only 20% of residents reported always counseling patients on diet for CV risk reduction.^12^ However, these findings cannot be generalized because the studies were conducted at single academic institutions.

Certain predictors of residents^’^ counseling practices have been identified, such as higher comfort levels and self-efficacy^13^; having a practice preference for primary care; and working with supervising physicians committed to prevention.^14^ However, no studies to date have examined the relationship between nutrition education in residency and residents’ counseling practices. Personal eating habits have also been shown to be predictor of counseling among medical students,^15^ but this association has not yet been examined among medical residents. Barriers to providing nutrition education have been examined among family medicine educators,^16^ but these findings may not be applicable to IM programs.

## Objectives and Hypotheses

Given the paucity of research on outpatient nutrition education during IM residency training, we piloted a nationwide needs assessment evaluating nutrition education and examining factors that might predict IM residents’ counseling practices for hypertension, hyperlipidemia, diabetes, and obesity. Our main hypothesis was that resident nutrition education (ie, amount of education and number of instruction methods used) would predict residents’ frequency of dietary counseling for patients. We also hypothesized that nutrition education would have stronger effects on counseling given a higher personal fruit and vegetable intake and residency program support for healthy eating habits. Finally, we explored barriers that educators face in providing nutrition education and barriers that residents face in counseling their patients.

## Methods

### Study design

We conducted a cross-sectional study using 2 structured online surveys, one for the program directors (PDs) and another for IM residents across the nation. We excluded combined programs, such as IM-Pediatrics or IM-Psychiatry and fellowships related to IM, to avoid heterogeneity of residency programs. We used the American Medical Association’s FREIDA online database of ACGME-accredited residency programs to obtain PDs’ contact information.^17^ Because the residents’ contact information was not publicly available, we requested the PDs to forward the survey to the residents. The survey was administered anonymously and confidentially, and the resident responses could not be matched to the PD responses and vice versa. The study was reviewed and approved by the University of California, Berkeley Institutional Review Board (protocol #2013-10-5737).

After a small pilot phase in December 2013, the surveys were administered nationally from January to February 2014, using Qualtrics software (Qualtrics LLC, Provo, UT, USA). Both surveys were designed to be completed in less than 10 minutes. We used phone calls and emails to the PDs to disseminate information and reminders about the study, and optional Amazon gift card raffles were used to increase participant response rate.

### Survey instrument

The surveys were developed based on previously published surveys used for medical students and residents.^18–21^ To inform the survey design, we used semistructured informal interviews with selected PDs at the lead author’s residency program, as well as interviews with residents from other programs. Appendix 1 shows the main measures in the survey instrument, response options, reliability, and validity information. Educators and residents were asked about the use of instruction modes for nutrition education at their institution. Residents were asked about the frequency of their counseling practices, perceived program support for healthy eating habits, and personal fruit and vegetable intake. In addition, the educators were asked about barriers to providing nutrition education to the residents, and the residents were asked about barriers they faced in providing dietary counseling.

### Statistical analysis

Inferential analyses were conducted using analyses of variance, *t* test, and χ^2^ test to examine associations between predictor variables (ie, amount of nutrition education and number of instruction methods used) and covariates as appropriate. For bivariate tests, the amount of education was summed across the 4 CV disease categories and then divided into “low” and “high” categories based on a median split. Similarly, the total number of instruction methods was collapsed into 2 categories (≤3 and >3) based on the identified median level of 3. The outcome variable (ie, residents’ frequency of dietary counseling) was summed across all 4 diseases and then condensed into 3 categories: “never/rarely,” “sometimes/half the time,” and “often/very often/always.”

For hypotheses testing, we first examined the outcome variable (ie, frequency of counseling) in relation to each predictor variable (ie, amount of nutrition education and number of instruction methods used) using linear regression techniques. Next, we examined the relationships between the outcome and the predictors using multivariable linear regression, controlling for confounders chosen based on prior literature review and bivariate associations from the descriptive analyses.

The 2 hypothesized moderators were program support for healthy eating and personal daily intake of fruits and vegetables. Responses across the 2 items assessing program support for healthy eating were summed. Similarly, responses across the 6 items assessing daily intake of fruits and vegetable items were summed. We tested moderation of the effect of each predictor variable by the moderators using interaction terms (eg, amount of education × fruit and vegetable intake, amount of education × program support for healthy eating habits) in multivariable analyses, and nonsignificant interaction terms were dropped.

For the barriers reported by the PDs, the “moderate” and “major” barriers were collapsed into one category. Similarly, the “important” and “very important” barriers reported by the residents were collapsed into one category. Pearson correlations were computed between the barriers reported by PDs and the predictor variables and between barriers reported by residents and frequency of counseling. We also calculated the frequencies of PDs’ and residents’ endorsement of barriers.

All analyses were conducted using Stata/IC 12.1 (StataCorp LLC, College Station, TX, USA), with exclusion of missing data. A *P* value of ≤.05 was used as the criterion for statistical significance.

## Results

### Sample characteristics of educators

A total of 40 educators (31 PDs and 9 associate PDs) responded out of the 393 eligible educators (response rate = 10.4% [40/393]), representing residency programs in 23 states. Most of the educators felt that nutrition education was moderately (41%) or somewhat (56.4%) important, but only 1 educator reported the presence of a formal curriculum on this topic at his or her program (Table 1). Less than 50% of the educators reported providing “quite a bit/extensive training in dietary counseling” on hypertension, hyperlipidemia, and obesity. The top 4 instruction methods for nutrition education were teaching by outpatient preceptors, teaching on inpatient wards, providing online material, and providing the residents a resource list of texts. The mean fruit and vegetable intake of the educators was 5.3 servings a day, and 60% reported 5 or more servings of fruits and vegetables per day.

**Table 1.** Sample characteristics of the educators and the Internal Medicine programs they belonged to.

		No. (%)
Respondent type	Program director	31 (77.5)
	Associate program director	9 (22.2)
Region	Northeast	11 (27.5)
	Midwest	7 (17.5)
	South	10 (25.0)
	West	12 (30.0)
Type of program	Community-based	5 (12.5)
	Community-based-university-affiliated	19 (47.5)
	University-based	14 (35.0)
	Other	2 (5.0)
Presence of primary care track	Yes	14 (35.0)
	No	26 (65.0)
% of residents entering primary care	0-20	22 (55.0)
	21-40	11 (27.5)
	41-60	5 (12.5)
	61-80	1 (2.5)
	81-100	1 (2.5)
Opinion on importance of nutrition education^a^	None	1 (2.6)
	Somewhat	16 (41.0)
	Moderately important	22 (56.4)
	Extremely important	0 (0.0)
Presence of formal curriculum^a^	Yes	1 (2.6)
	No	38 (97.4)
Reported providing “quite a bit”/“extensive” training in dietary counseling for^b^	Obesity	16 (42.1)
	Hypertension	18 (47.4)
	Dyslipidemia	18 (47.4)
	Diabetes	20 (52.6)
Methods used to teach^b^	Teaching by preceptors in primary care clinic	36 (95.0)
	Teaching on inpatient wards	30 (79.0)
	Providing online material	30 (79.0)
	Providing resource list of texts	23 (60.5)
	Participating in specialty clinic that focusses on nutrition	15 (40.0)
	Scholarly projects (eg, quality improvement/curricula improvement)	14 (37.0)
	Elective offering	11 (29.0)
	Structured individual study with selected reading material	8 (21.1)
	Other	4 (10.5)
	Structured individual study with educational CD	1 (2.6)
	Attendance at a national nutrition conference	1 (2.6)
Total fruit and vegetable intake (mean ± SD)		5.3 ± 2.8
≥5 servings of fruit and vegetable intake a day		24 (60)

a 1 educator with missing information.

b 2 educators with missing information.

### Sample characteristics of residents

A total of 133 IM residents from 19 states took the survey. Approximately 10% of the residents reported receiving nutrition education via a formal curriculum, and 61% of the residents reported having none or little bit of training in nutrition across the 4 CV risk factors. The median number of instruction methods was 3, ranging from 0 to 7. The most frequently used instruction methods were the same as those reported by the PDs. A total of 38% of residents reported counseling their patients “none of the time” or “rarely,” 48% reported counseling “half the time,” and 22% reported counseling “often or always.” Furthermore, 61% of residents agreed or strongly agreed that their program encouraged healthy eating habits, and 55% of residents agreed or strongly agreed that their program provided healthy meal options. The mean fruit and vegetable intake of the residents was 3.2 servings a day, and 32% reported 5 or more servings of fruits and vegetables per day.

### Resident characteristics by nutrition education received and frequency of dietary counseling

Residents were more likely to report a higher amount of nutrition training if they belonged to a program in the Northeast and Western regions (vs Midwest and Southern regions), if their program was a community-based program (vs a university-affiliated community program or a university-based program), and if they went to medical school abroad (vs medical school in the United States). Similarly, residents were significantly more likely to report more than 3 instruction methods if they were older, if they belonged to a program in the Northeast or West (vs Midwest and Southern regions), if they belonged to a community-based program (vs community-based-university-affiliated and university-based programs), and if they had any nutrition education (vs none) before medical school (Table 2). Residents reported counseling their patients more frequently if their program was in the Northeast or Midwest (vs West and Southern regions) and if their program had a primary care track (vs not) (Table 3).

**Table 2.** Resident sample characteristics by the number of methods used to learn about nutrition for the outpatient setting.

	No. of methods		*P* value
	≤3	>3	
No. (%)^a^	70 (56.0)	55 (44.0)	
Age (y, mean ± SD)	29 ± 3	30 ± 3	**.02**
Gender (n)			.94
Female	39 (55.7)	31 (44.3)	
Training level (n)			.47
Post graduate year 1	29 (55.8)	23 (44.2)	
Post graduate year 2	22 (64.7)	12 (35.3)	
Post graduate year 3	12 (44.4)	15 (55.6)	
Post graduate year 4	7 (58.3)	5 (41.7)	
Career path (n)			.89
Primary care	19 (57.6)	14 (42.4)	
Subspecialty	35 (53.0)	31 (47.0)	
Undecided	10 (62.5)	6 (37.5)	
Other	6 (60.0)	4 (40.0)	
Region (n)			**.05**
Northeast	23(49.0)	24 (51.0)	
Midwest	16 (66.7)	8 (33.3)	
South	21 (72.4)	8 (27.6)	
West	9 (39.1)	14 (60.8)	
Type of program (n)			**<.001**
Community-based	8 (24.2)	25 (75.8)	
Community-based-university-affiliated	29 (63.0)	17 (40.0)	
University-based	33 (71.7)	13 (28.3)	
Presence of PC track (n)			.24
Yes	44 (52.4)	40 (47.6)	
No	26 (63.4)	15 (36.6)	
In PC track (of those in programs with a PC track) (n)			.14
Yes	11 (40.7)	16 (59.3)	
No	33 (57.9)	25 (42.1)	
Medical education (n)			.09
US	52 (61.2)	33 (38.8)	
Foreign	18 (45.0)	23 (55.0)	
Prior nutrition education (n)			
Before medical school	16 (76.2)	6 (23.8)	**.04**
In medical school	42 (53.2)	39 (46.8)	**.33**
Daily fruit and vegetable intake (mean no. of servings ± SD)	3.6 ± 2.6	4.9 ± 4.9	

Abbreviation: PC, primary care.

a 8 residents with missing information.

Bold values in the table represents numbers which are statistically significant.

**Table 3.** Resident sample characteristics by frequency of nutrition counseling in the outpatient setting.

	Never/rarely	Sometimes/half the time	Often/very often/always	*P* value
No. (%)^a^	38 (32.7)	56 (48.3)	22 (19.0)	
Age (y, mean ± SD)	28.9 ± 2.2	29.6 ± 3.2	30.4 ± 2.7	.13
Gender (n)				.51
Female	23 (35.4)	32 (49.2)	10 (15.4)	
Training level (n)				.53
Post graduate year 1	16 (34.0)	21 (44.7)	10 (21.3)	
Post graduate year 2	13 (39.4)	16 (48.5)	4 (12.1)	
Post graduate year 3	6 (24.0)	15 (60.0)	4 (16.0)	
Post graduate year 4	3 (27.2)	4 (36.4)	4 (36.4)	
Career path (n)				.38
Primary care	8 (25.0)	21 (65.6)	3 (9.4)	
Subspecialty	22 (36.1)	25 (41.0)	14 (22.9)	
Undecided	5 (33.3)	6 (40.0)	7 (26.7)	
Other	3 (37.5)	4 (50)	1 (12.5)	
Region (n)				**.01**
Northeast	9 (20.4)	24 (54.6)	11(25.0)	
Midwest	3 (13.0)	15 (65.2)	5 (21.7)	
South	14 (53.9)	9 (34.6)	3 (11.5)	
West	11 (52.4)	7 (33.3)	3 (14.3)	
Type of program (n)				.20
Community-based	8 (26.7)	14 (46.6)	8 (26.7)	
Community-based-university-affiliated	17 (38.7)	17 (38.7)	10 (22.7)	
University-based	13 (31.0)	25 (59.5)	4 (9.5)	
Presence of PC track (n)				**.03**
Yes	20 (25.6)	44 (56.4)	14 (18.0)	
No	18 (47.4)	12 (31.6)	8 (21.0)	
In PC track (n)				.66
Yes	5 (19.2)	16 (61.5)	5 (19.2)	
No	15 (28.9)	28 (53.8)	9 (17.3)	
Medical education (n)				.10
US	29 (36.7)	39 (49.4)	11 (13.9)	
Foreign	9 (24.3)	17 (46.0)	15 (29.7)	
Prior nutrition education (n)				
Before medical school	8 (42.1)	8 (42.1)	3 (15.8)	.64
In medical school	20 (26.3)	41 (54.0)	20 (19.7)	.12
Daily fruit and vegetable intake (mean no. of servings ± SD)	3.4 ± 2.1	4 ± 2.4	7.5 ± 6.7	.45

Abbreviation: PC, primary care.

a 12 residents with missing information.

Bold values in the table represents numbers which are statistically significant.

### Predictors of frequency of dietary counseling

In the unadjusted linear regression analyses, 2 key predictors (ie, amount of education received and number of instruction methods used) were positively associated with frequency of counseling patients (β = 0.39 and 0.43, respectively, *P* < .001) (Table 4). As hypothesized, these 2 predictors remained positively associated with frequency of patient counseling (β = 0.20, *P* = .05; β = 0.26, *P* = .02) after adjusting for confounders listed in the table. In addition, total fruit and vegetable intake (β = 0.24, *P* < .001) and nutrition education in medical school (β = 0.20, *P* = .03) were positively associated with frequency of counseling (Table 4).

**Table 4.** Bivariate and multivariate predictors of residents’ frequency of dietary counseling in the outpatient setting.

	β^a^	SE	*P* value
Bivariate linear regression			
Amount of training	0.39	0.18	**<.001**
No. of methods	0.43	0.06	**<.001**
Multivariable linear regression			
Amount of training	0.20	0.21	**.05**
No. of methods	0.26	0.08	**.02**
Total fruit and vegetable intake	0.24	0.03	**<.001**
Nutrition education in medical school	0.20	0.24	**.03**
Post graduate level	0.19	0.12	.06
Age	−0.05	0.04	.62
Gender	0.09	0.21	.31
Path	0.13	0.14	.15
Type of program	−0.10	0.17	.32
Presence of primary care track	0.03	0.23	.72
Being in primary care track	0.08	0.31	.40
Medical education in the United States	−0.03	0.28	.78
Nutrition education before medical school	0.10	0.31	.28
Region			
Northeast (as reference)			
Midwest	0.36	0.30	.71
South	−0.16	0.31	.13
West	−0.16	0.32	.12

a A standardized β coefficient was used to account for differences in units of the variables.

Bold values in the table represents numbers which are statistically significant.

Contrary to expectations, personal fruit and vegetable intake, healthy meal provision by residency programs, and program support for residents’ healthy eating habits did not significantly moderate the relationships between the 2 predictor variables and the frequency of dietary counseling. Interaction terms were thus dropped.

### Barriers faced by educators in providing nutrition education

Pearson correlation tests showed that lack of faculty expertise was associated with using fewer instruction methods used in the program (*r* = −.33, *P* = .04) (Table 5). The most frequently endorsed moderate-to-major barriers were competing curricular demands, lack of physician faculty with expertise in nutrition, inadequate financial resources, and lack of administrative support (Table 5).

**Table 5.** Barriers faced by program directors in providing nutrition education.

Barrier	Correlation with no. of methods used	*P* value	Correlation with amount of training provided	*P* value	% reporting moderate-to-major barrier
Lack of physician faculty with expertise in nutrition	−0.33	**.04**	−0.13	.45	76
Lack of faculty interest	−0.17	.33	0.03	.83	54
Competing curricular demands	−0.15	.36	−0.22	.19	80
Unclear evidence base for nutrition interventions	−0.11	.53	0.21	.20	33
Lack of ACGME requirement	−0.09	.59	0.10	.55	26
Lack of administrative support	−0.08	.63	0.05	.76	61
Lack of resident interest	0.06	.69	0.21	.20	43
Inadequate financial resources for program development	−0.06	.73	0.12	.47	61
Other	0.02	.87	−0.22	.18	22
1. “Teaching nutrition takes time”					
2. “Work flow challenges”					
Lack of insurance reimbursement for nutrition interventions	0.008	.96	−0.04	.81	48

Bold values in the table represents numbers which are statistically significant.

### Barriers faced by residents in dietary counseling

Even though endorsed by a minority of residents, lack of personal interest in providing dietary counseling and perceived lack of clinic preceptors’ interest in nutrition were associated with lower frequency of counseling (*r* = −.19, *P* = .04; *r* = −.18, *P* = .05, respectively; Table 6). The most frequently endorsed important barriers were lack of time, perception that patients were coming in for a different purpose, and perceived lack of patient interest in nutrition (Table 6).

**Table 6.** Barriers faced by residents in counseling patients on diet.

Barrier	Correlation with counseling provided	*P* value	% reporting as important/very important
Lack of personal interest in providing nutrition counseling	−0.19	**0.04**	21
Lack of clinic preceptor’s interest in nutrition	−0.18	**0.05**	31
Lack of proper patient education materials	−0.18	0.06	45
Patients come for a different purpose	−0.17	0.08	59
Lack of availability of health educators	−0.13	0.16	45
Insufficient reimbursement	−0.13	0.17	33
Lack of time	−0.12	0.20	69
Lack of patient interest in nutrition	0.08	0.40	52
Lack of systems for tracking and prompting nutrition counseling	−0.05	0.55	46
Cultural differences between you and your patients	0.01	0.91	25

Bold values in the table represents numbers which are statistically significant.

## Discussion

In this contemporary assessment of nutrition education in IM residency training among 40 educators and 133 residents across the United States, most of the residents reported insufficient training in dietary counseling for CV risk factors such as hypertension, dyslipidemia, diabetes, and obesity. Multimodal nutrition education during residency independent of nutrition education in medical school as well as personal fruit and vegetable intake was found to be predictors of residents’ frequency of counseling their patients. Educators lacked expertise in teaching nutrition and faced competing curricular demands in providing nutrition education. Residents in training faced lack of personal and supervising faculty interests as barriers to counseling their patients.

### Nutrition education in residency

Over the past 2 decades, studies have consistently shown that nutrition education is valued in the medical community, but not adequately covered in medical training.^3,6^ Our study confirms this alarming finding for IM across the United States, the specialty that produces the largest number of physicians each year,^7^ most of whom will see patients in the outpatient setting (primary care or subspecialty).^22^ Most of the residents reported having none or only a little training in nutrition counseling for the 4 diet-related CV risk factors. Furthermore, only 1 IM program in our study provided a formal curriculum in nutrition education; however, this may be a conservative estimate due to our small sample size.

Several teaching instruction methods, other than a formal curriculum, have been recently proposed to teach nutrition during residency. These range from brief immersion courses to longitudinal exposure and from required rotations to optional online modules.^23^ Contrary to these new proposals and older studies,^9,10^ we found that currently the most frequently used instruction methods in IM programs are resource list of texts, preceptor teaching in the outpatient setting, inpatient teaching, and online sources. We found differences by region and type of residency program in the utilization of multimodal education by the residents, which need to be explored further.

### Residents’ dietary counseling practices

Our study found that only 22% of residents reported counseling their patients often/always on dietary changes, similar to the low proportion (20%) reported 10 years ago.^12^ To our knowledge, this is the first study conducted across multiple programs in the United States that provides evidence for the need to improve contemporary nutrition education in IM residency programs. We showed that nutrition education in residency, independent of education in medical school, is associated with higher frequency of dietary counseling by residents. Nutrition education in medical school was also a predictor of counseling practices, but based on the effect sizes as shown in the results, multimodal nutrition education in residency may be a more important predictor of residents’ counseling practices, reflecting recent discussions among medical educators.^24^ This finding supports use of multiple educational strategies for nutrition education in residency programs similar to the longitudinal nutrition education approach that has been proposed for the medical school curriculum.^25^

In addition to receiving more nutrition education, we found that residents with better personal dietary habits (ie, fruit and vegetable intake) also counseled their patients on dietary changes more frequently. Several studies have reported associations between practicing physicians’ health and patient counseling outcomes.^26–28^ Our study is among the first to explore residents’ dietary habits using a validated screening tool.^20^ We found that residents had a lower mean intake of fruits and vegetables per day than their educators. Although most of the educators met the daily requirement of 5 servings, only a minority of residents met this requirement. Reassuringly, we found that most residents felt that their programs provided healthy meal options and encouraged a healthier diet. Therefore, other factors, such as high stress levels, need to be explored to explain residents’ unhealthy eating habits and to identify ways to promote a healthier diet among the residents.

### Barriers

Consistent with an older study from the 1990s,^3^ educators’ responses indicated that lack of physician faculty expertise remains a key barrier to nutrition education in residency programs today. In addition, and similar to previous studies,^16,18^ competing curricular demands, inadequate financial resources, and lack of administrative support were found to be major barriers for IM educators in our study. However, contrary to an older study,^3^ we did not find that lack of evidence for nutrition interventions or inadequate reimbursement for dietary counseling was a major barrier to providing nutrition education. These findings are reassuring given the strong evidence for dietary changes in CV risk management.^2^ Thus, accreditation organizations should consider supporting institutional reform by including prevention and nutrition education in IM residency curricula, which would then prioritize financial and administrative resources for nutrition education and ultimately generate greater physician faculty expertise.

Regarding counseling patients on diet, we found that lack of personal and perceived clinic preceptor’s interests in nutrition were associated with less nutrition counseling by residents. Hence, while residents and educators may not have control over lack of time and patient factors, we speculate that by starting a culture of nutrition education in residency programs, we can increase interest in this area. Interestingly, lack of health educators was not one of the top barriers reported by the residents. This may be because the respondents belonged to programs that were likely to have support from registered dieticians/health educators already. Nonetheless, per national labor statistics, for every 24 physicians, only 1 dietician is available,^29,30^ so expecting dieticians alone to provide dietary counseling would be unrealistic.

Our study has several limitations. First, despite extensive efforts to ensure a high response rate, the response rate of the PDs remained low. Also, because resident participation was dependent on the PDs forwarding the survey to residents, we could not determine the number of residents that received the survey link or calculate resident response rate. However, our study could serve as a pilot for more formal and systematic data collection (eg, through accreditation organizations) to capture all residency training programs in the United States.

Second, the resident sample was not representative of the national pool of residents, limiting the generalizability of our findings. Although we did receive resident responses from 19 states, the Western United States was overrepresented. Furthermore, comparison of the characteristics of our resident sample with those of the national pool of residents^31^ showed that while the average ages were similar (29 vs 29.2 years), our sample included higher proportions of women (56% vs 40%) and foreign medical graduates (42% vs 32%). Similarly, while only 13% of IM programs nationally were designated as “Medicine-Primary” in 2014,^7^ in our study, 35% of the programs had a primary care track based on educators’ responses. As prior literature^14^ demonstrates that female physicians, foreign medical graduates, and residents interested in primary care report higher dietary counseling rates, a nationally representative sample may show even lower counseling rates than our results. In addition, we speculate that the *relationships* between nutrition education and dietary counseling reported here would likely hold in a representative sample. Nonetheless, future research would need to confirm these findings in larger and representative samples.

Third, as with any study using self-reported survey data not verified by chart audits or observation, the results may be subject to error due to recall, selection, social desirability biases, and such. Similarly, we could not assess the quality of the dietary counseling provided, which may be a better predictor of patient outcomes than the frequency of dietary counseling.

Finally, our cross-sectional study cannot establish a causal association between nutrition education and patient counseling. However, our multivariable analyses did comprehensively address potential confounders, including nutrition education in medical school, thus supporting the need to improve nutrition education in graduate medical training.

## Conclusions

The provision of nutrition education in IM residency programs and IM residents’ dietary counseling for patients need to be systematically assessed nationally (eg, through accreditation organizations). The preliminary findings of this study suggest that multimodal nutrition education in IM residency and better resident dietary habits are associated with higher frequency of dietary counseling for patients. Faculty expertise and faculty and resident interests in patient counseling could be improved perhaps by mandating nutrition education in graduate and continuing medical education. Future studies need to replicate current findings in large, representative samples; assess quality of dietary counseling; and examine how different instructional modes relate to resident learning and patient outcomes.

## Appendix

**Appendix 1.** Survey instrument and variable treatment.

Variable		Item	Answer options
Predictor variables	Amount of nutrition education across each: obesity, diabetes, hypertension, dyslipidemia	“How much training have you had on dietary counseling for the following diseases in the outpatient setting?”	“None at all,” “a little bit,” “quite a bit,” and “extensive”
	No. of instruction methods^18^	“How have you learned about outpatient nutrition and dietary counseling for cardiovascular risk factors (obesity, hypertension, dyslipidemia, diabetes) during residency?”	“Teaching by preceptors in primary care clinic” “Teaching on inpatient wards” “Providing online material” “Providing resource list of texts” “Participating in specialty clinic that focusses on nutrition. Specify”: “Scholarly projects (eg. quality improvement/curricula improvement) Elective offering, Specify”: “Structured individual study with selected reading material” “Structured individual study with educational CD” “Attendance at a National nutrition conference” “Other, Specify”
Outcome variable	Self-reported frequency of counseling^a^	“In a typical ambulatory week, for what percentage of patients with the following cardiovascular risk factors, do you engage in dietary counseling?”	“Never (0%),” “rarely (1%-20%),” “sometimes (21%-40%),” “half the time (41%-60%),” “often (61%-80%),” “very often (81%-99%),” and “always (100%)”
Moderators	Program support for healthy eating habits^b^	“Residency sponsored meals have healthy food options” “My residency program encourages me to eat healthy”	“Strongly disagree,” “disagree,” “agree,” and “strongly agree”
	Personal daily fruit and vegetable intake^c^	Diet screener with 6 items: fruit juice, fruit other than as juice, vegetable juice, green salad, vegetable soup or stew, any other vegetables	No. of servings per day ranging from 0 to 6 and per day/week/month

a Adapted from the Preventive Medicine Attitudes and Activities Questionnaire (PMAAQ) scale (α = .85, test-retest reliability correlation = 0.72).^19^

b Two-item scale had an α of .78.

c Validated in medical students with reproducibility correlation *r* = .77 and correlation with Food Frequency Questionnaire *r* = .50.^20^
